# Performance Comparisons of AlexNet and GoogLeNet in Cell Growth Inhibition IC50 Prediction

**DOI:** 10.3390/ijms22147721

**Published:** 2021-07-19

**Authors:** Yeeun Lee, Seungyoon Nam

**Affiliations:** 1 Department of Health Sciences and Technology, Gachon Advanced Institute for Health Sciences and Technology, Gachon University, Incheon 21999, Korea; soirlechat@gachon.ac.kr; 2 College of Medicine, Gachon University, Incheon 21565, Korea; 3 Gachon Institute of Genome Medicine and Science, Gachon University Gil Medical Center, Incheon 21565, Korea; 4 Department of Life Sciences, Gachon University, Seongnam 13120, Korea

**Keywords:** pharmacogenomics, drug responsiveness, deep learning, machine learning

## Abstract

Drug responses in cancer are diverse due to heterogenous genomic profiles. Drug responsiveness prediction is important in clinical response to specific cancer treatments. Recently, multi-class drug responsiveness models based on deep learning (DL) models using molecular fingerprints and mutation statuses have emerged. However, for multi-class models for drug responsiveness prediction, comparisons between convolution neural network (CNN) models (e.g., AlexNet and GoogLeNet) have not been performed. Therefore, in this study, we compared the two CNN models, GoogLeNet and AlexNet, along with the least absolute shrinkage and selection operator (LASSO) model as a baseline model. We constructed the models by taking drug molecular fingerprints of drugs and cell line mutation statuses, as input, to predict high-, intermediate-, and low-class for half-maximal inhibitory concentration (IC50) values of the drugs in the cancer cell lines. Additionally, we compared the models in breast cancer patients as well as in an independent gastric cancer cell line drug responsiveness data. We measured the model performance based on the area under receiver operating characteristic (ROC) curves (AUROC) value. In this study, we compared CNN models for multi-class drug responsiveness prediction. The AlexNet and GoogLeNet showed better performances in comparison to LASSO. Thus, DL models will be useful tools for precision oncology in terms of drug responsiveness prediction.

## 1. Introduction

Precision medicine has been developed to provide optimized diagnosis and therapeutic treatment for individual patients toward better treatment results [1]. For this purpose, the genetic information of patients can maximize the effects [2,3]. It is essential to study drug responsiveness according to the genomic profiling of each patient. Testing drugs in real patients for drug responsiveness, however, is not allowed ethically. Therefore, with the development of large-scale pharmacogenomics databases, precision medicine becomes reality [4,5,6]. Thus, to study drug responsiveness in diseases, pharmacogenomics data have been critical [7,8]. These databases included cell line genomic profiles of drug treatments, and drug responsiveness data in the cell lines [4,8,9,10,11,12].

Cancer is a dynamic disorder caused by the accumulation of mutations, resulting in genetic heterogeneity of cancer cells [13,14,15,16,17]. As a result of this heterogeneity, even in the same cancer type, patients have their own different mutation profiles, presumably leading to different sensitivity to treatments [14]. Therefore, individualized therapies for patients are of paramount importance [18]. Thus, cancer is a representative disease that requires precision medicine [6].

Currently, most of the drug responsiveness prediction methods for cancer have used the pharmacogenomics databases to identify the correlation (e.g., positive- and anti-correlation) between the genomic profiles of cancer cell lines and individual cancer patients. For example, these methods, in general, predict a drug such that the gene expression profiles after the drug exposures are negatively correlated with the gene expression profiles of an individual patient [8,19]. So far, machine learning (ML) approaches have also been used for drug responsiveness prediction [12,20].

Currently, deep learning (DL) approaches are used for drug responsiveness prediction in the field of cancer. There are a dozen of DL approaches [21,22,23,24,25,26,27,28] for predicting drug responsiveness. Most of these approaches accepted AlexNet-based convolution neural networks (CNNs) [28] and autoencoders [23]. Autoencoder-based models included DeepAEs [25], Dr.VAE [23], and DeepDSC [21]. CNN-based models included tCNNs [29], KekuleScope [30], and CDRscan [31]. In the model construction, training data sources included Connectivity Map (CMap) [32], the Cancer Cell Line Encyclopedia (CCLE) [33], Genomics in Drug Sensitivity in Cancer (GDSC) [34], the Catalogue of Somatic Mutations in Cancer (COSMIC) [35], and the Genomic Data Commons Data Portal (GDC) [36]. Those DL approaches demonstrated good accuracy in comparison to traditional ML approaches [37,38]. Those DL approaches have the potential to support contemporary precision-medicine endeavors and relevant clinical decision-making tasks. These approaches take cancer cell line genomic profiles and chemical properties of treated drugs in the cell line as input. Then, these approaches feed the genomic profiles of individual cancer patients into the DL models for drug responsiveness prediction [28].

AlexNet, one of CNN architectures, was introduced for predicting multi-class drug responsiveness prediction by using the pharmacogenomics databases [28]. Previous studies [39,40] demonstrated that the deeper or more layers the model, the higher the accuracy. In fact, several attempts of CNN models with many layers showed generally high performances. However, the performance does not increase perfectly in proportion to the number of layers, due to the vanishing gradient problem in which the gradient gets smaller during backpropagation, keeping the weight from updating its value [40]. GoogLeNet was introduced as an attempt to solve the problem [41]. However, other CNN architectures such as GoogLeNet, in particular, in the multi-class drug responsiveness prediction DL models have not been compared with AlexNet [6,8,14,16,21,28,42,43,44,45].

In this study, we compared CNN architectures GoogLeNet [41] and AlexNet [28] for drug responsiveness prediction, by training pharmacogenomics data. DeepIC50 was used for AlexNet, and GoogLeNet was implemented for this study. We trained the two CNN models by using genomic profiles of cancer cell line and chemical structural data of drugs as features, to predict drug responsiveness defined by half maximal inhibitory concentration (IC50) of drug exposure. The least absolute shrinkage and selection operator (LASSO) model was used as a ML baseline model [46,47,48]. Our problem is a multi-class prediction by categorizing the drug responsiveness values (i.e., IC50 values) into three classes. Thus, the micro-average area under receiver operating characteristic (AUROC or AUC) curves, and the macro-average AUROCs of the three models were compared. [46,47,48,49]. When tested with an independent gastric cancer (GC) cell line dataset [33], GoogLeNet [41] and AlexNet [28] had higher accuracy than the baseline LASSO model. Moreover, AlexNet showed better accuracy than GoogLeNet. We also applied the three models to another independent dataset, clinical response data of chemotherapeutic agents, and genomic profiles of individual patients in The Cancer Genome Atlas (TCGA) breast cancer dataset (henceforth, TCGA-BRCA) [36].

## 2. Results

### 2.1. Overview

**Figure 1:**
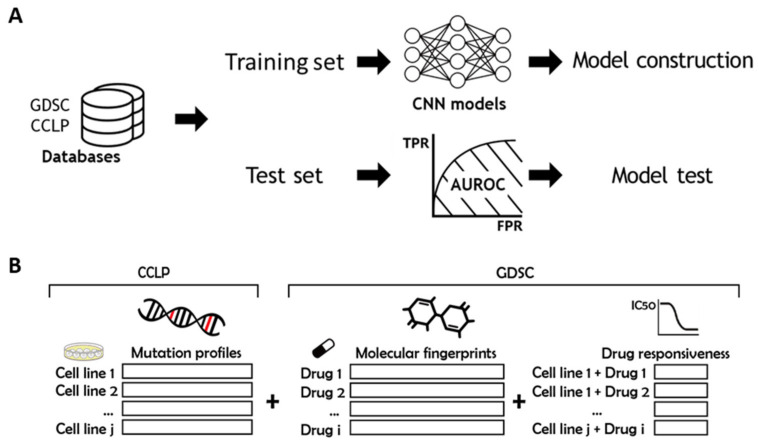
Overview of scheme. (**A**) To build and train two CNN models, and the LASSO baseline model, pharmacogenomics data from CCLP [35] and GDSC [34] were used. Independent GC cell line data from CCLLE [33] were used to test the performance of the three models in predicting drug responsiveness. The performance of the three models was compared by AUROCs. (**B**) Mutation profiles data for cancer cell lines were obtained from CCLP. Molecular fingerprints data for drugs and responsiveness data for ‘cancer cell lines and drugs’ were obtained from GDSC. The model was trained to predict drug responsiveness by calculating mutation profiles data and molecular fingerprints data as features.

To compare the prediction performance of two CNN models using the half-maximal inhibitory concentration (IC50) value of a drug for cancer cell lines, we obtained the genomic mutation dataset, drug molecular properties, and *ln* (IC50) values from GDSC and CCLE. Subsequently, we divided the data from GDSC into training set and test set, and then, we compared the performance of two CNN models and the baseline model using micro- and macro-average AUROCs (Figure 1).

### 2.2. Model Construction and Performance of GoogLeNet

**Figure 2:**
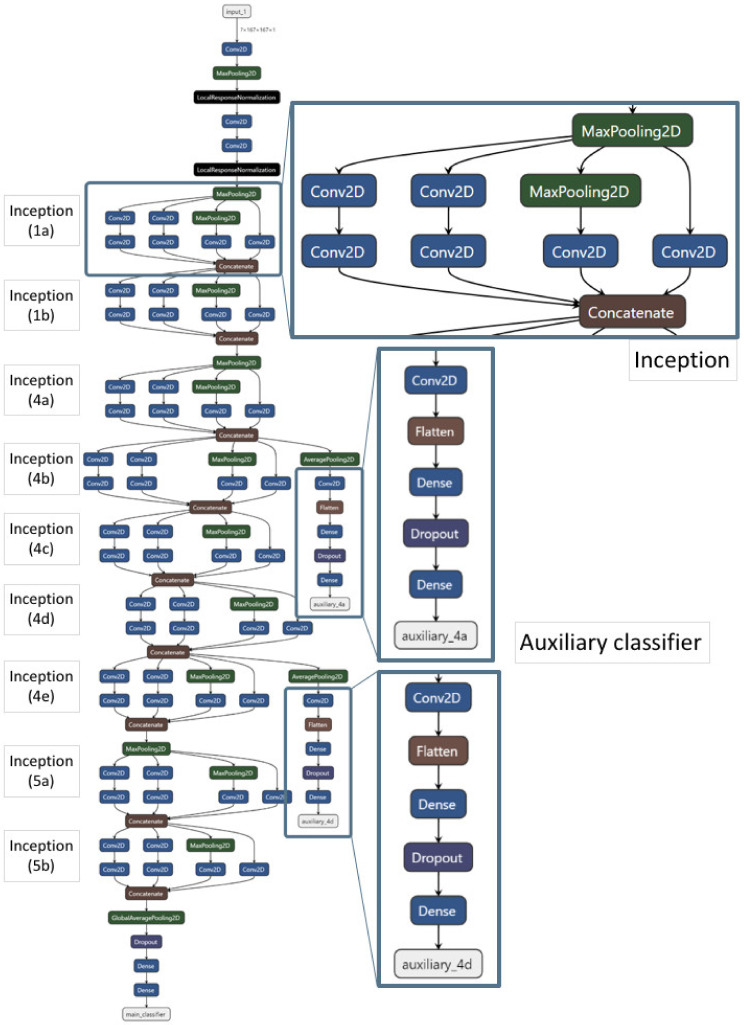
Model structure of GoogLeNet. It contained several 2-dimensional convolution layers (Conv2D), 2-dimensional max pooling layers (MaxPooling2D), 2-dimensional average layers (GlobalAveragePooling2D), Inception-v1 modules (the upper box), and two auxiliary classifiers (two lower boxes).

**Figure 3:**
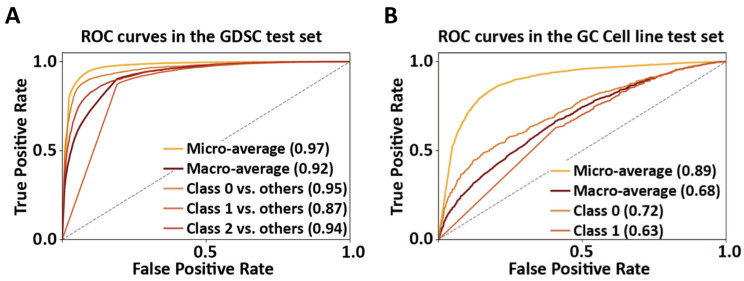
GoogLeNet model performance on GDSC test set and GC cell line test set. Micro-/macro-average and each class’ OVR AUROCs are depicted. The graphs represent the performance of GoogLeNet in the GDSC test set (**A**), and in the GC cell line test set (**B**). It is noted that the GC cell line test set, from CCLE, did not contain class 2 cases. The numerals in parentheses indicate the AUROCs.

The GoogLeNet model was constructed with Inception-v1 modules and auxiliary classifier (Figure 2), and subsequently trained by using the GDSC training set. In the GDSC test set, micro-average AUROC was 0.97, and macro-average AUROC 0.92. The AUROCs for one-versus-rest (OVR) classifications were 0.95 for class 0 vs. other classes; 0.87 for class 1 vs. other classes; and 0.94 for class 2 vs. other classes; Overall, the AUROCs for class 1 (i.e., intermediate drug responsiveness) was relatively low, but for class 0 and class 2, they were high (Figure 3A).

In another independent GC cell line test set, the micro-average AUROC was 0.89 and the macro-average AUROC 0.68. The AUROCs of OVR classifications were 0.72 for class 0 vs. other classes; and 0.63 for class 1 vs. other classes (Figure 3B).

### 2.3. Performance Comparisons of GoogLeNet, DeepIC50, and LASSO

**Figure 4:**
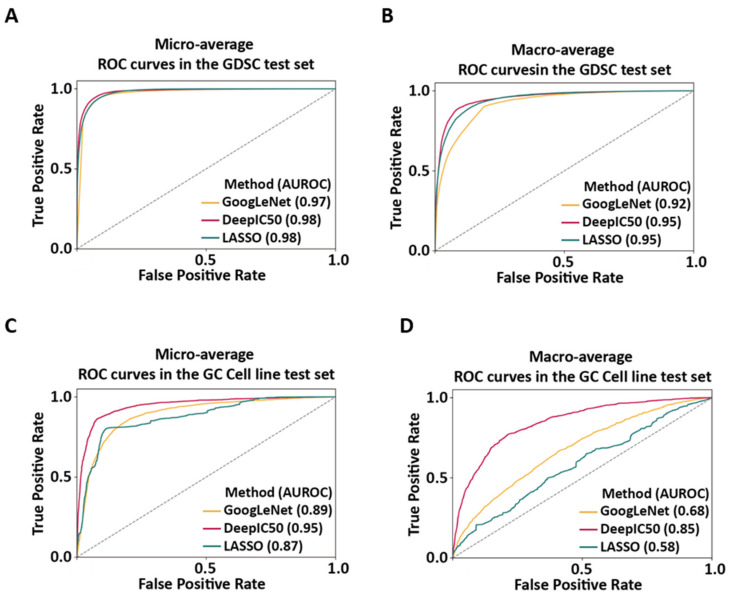
Comparison of GoogLeNet and DeepIC50 along with the baseline LASSO model. The prediction performances of the two CNN models and LASSO were compared by drawing micro-/macro-average ROC curves. (**A**) The micro-average AUROCs in the GDSC test set. (**B**) The macro-average AUROCs in the GDSC test set. (**C**) The micro-average AUROCs in the GC cell line test set. (**D**) The macro-average AUROCs in the GC cell line test set.

With the same GDSC training set, GoogLeNet, DeepIC50, and LASSO models were trained. Then, the performances were measured in the GDSC test set and the GC cell line test set, and the performance of each model was compared. In the GDSC test set, GoogLeNet showed micro-average AUROC of 0.97 and macro-average AUROC of 0.92; DeepIC50 micro-average AUROC of 0.98 and macro-average AUROC of 0.95; and LASSO model micro-average AUROC of 0.98 and macro-average AUROC of 0.95 (Figure 4A,B). Since our problem was the three-class classification, the same OVR approach for GoogLeNet, DeepIC50, and LASSO was applied to obtain the Matthew Correlation Coefficients (MCCs) in the GDSC test set. The three models’ MCCs were comparable in classifying class 0 and the other classes, while the two CNN models were better over LASSO in classifying not only class 1 and the other classes, but also class 2 and the other classes (Table S1). In the independent GC cell line test set, GoogLeNet showed micro-average AUROC of 0.89 and macro-average AUROC of 0.68; DeepIC50 showed micro-average AUROC of 0.95 and macro-average AUROC of 0.85; and the LASSO model showed micro-average AUROC of 0.87 and macro-average AUROC of 0.58 (Figure 4C,D). Both CNN models, DeepIC50 and GoogLeNet, showed better performances than the LASSO baseline model in the GDSC test set (Table S2) and the GC cell line test set (Table S3).

### 2.4. Application of GoogLeNet, DeepIC50, and LASSO to the TCGA-BRCA Patient Dataset

The models were also compared in another test set, clinical follow-up data in TCGA-BRCA. GoogLeNet showed micro-average AUROC of 0.62 and macro-average AUROC of 0.51 (Figure S1A). DeepIC50 reported micro-average AUROC of 0.56 and macro-average AUROC of 0.44 (Figure S1B,C). LASSO demonstrated micro-average AUROC of 0.59 and macro-average AUROC of 0.42 (Figure S1B,C). Overall, the performance of GoogLeNet among the three models was best, and their performances in the real patients were marginal. The confusion matrix for GoogLeNet in the patient dataset was represented in Table S4.

**Figure 5:**
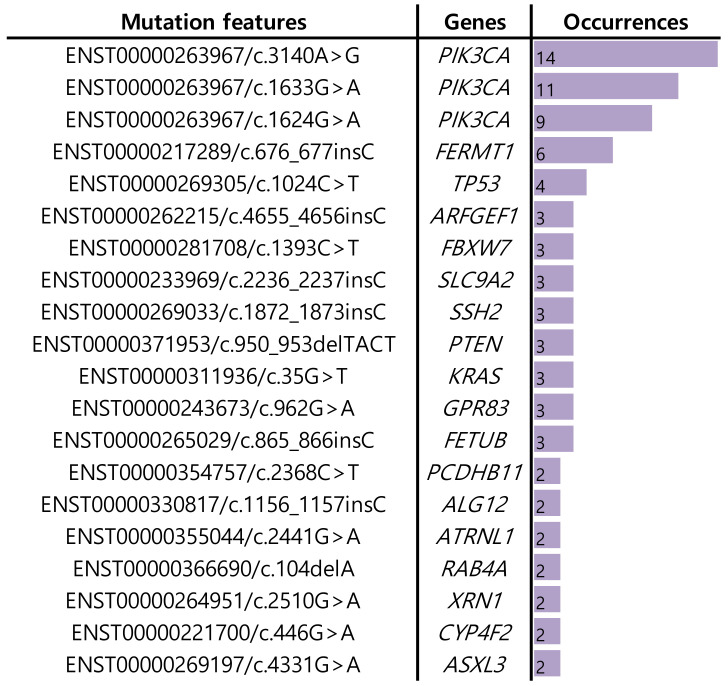
Important features of GoogLeNet, by LIME, in the TCGA-BRCA dataset. Column ‘occurrences’ in a given mutation feature indicates the frequency observed in the 322 patients by LIME.

To identify important features in GoogLeNet, we utilized the local interpretable model-agnostic explanation (LIME) [50]. From a total of the 322 patients, 100 explainable features for each patient were obtained, and, out of the features, we selected 20 abundant mutation features among the patients (Figure 5). Genes including *PIK3CA, FERMT1*, and *TP53* were obtained.

## 3. Discussion

In this study, we compared CNN models, AlexNet [28] and GoogLeNet [41], for three-class classification of drug responsiveness, by using mutation statuses of cell lines and molecular properties (including molecular fingerprints) of drugs. Through the comparisons of the ML baseline model and the two CNN models, the CNN models showed better performances over the ML model. Moreover, AlexNet-based DeepIC50 demonstrated better performance for multiple-class responsiveness prediction over the other two models in the GDSC test set and the GC cell line test set. In the next section, we describe utility of DL-based models.

Recently, DL-based drug responsiveness prediction models have emerged [26]. DL models of drug responsiveness prediction have been built in large-scale pharmacogenomics databases from the disease model systems. This is largely because measuring drug responsiveness by treating patients is not possible due to ethical concerns. Alternately, cell line experiments are accepted, as proxies for cancer patients. Thus, in the case of our study, after building drug responsiveness models based on the pharmacogenomics databases containing drug treatments in cancer cell lines, we took a concatenation vector of a mutation status vector of a patient and a drug chemical property vector as input for the models. Subsequently, these models could predict the responsiveness to a drug for the patient.

We applied this strategy to a real breast cancer patient dataset, TCGA-BRCA, which contained clinical follow-up information for chemotherapeutic agents. In the TCGA-BRCA dataset, we summarized AUROC differences of the two nets, in contrast with LASSO (Figure S1B,C and Table S5), indicating better performance of GoogLeNet over DeepIC50. In particular, the micro-average AUROCs of LASSO and DeepIC50 were closer to 0.5, in comparison to GoogLeNet (Figure S1B), indicating that LASSO and DeepIC50 made random guesses. The breast cancer (BRCA) is considered as one of the most heterogenous tumor [51], indicating that the cancer differs greatly among the cancer patients [52]. Even patients belonging to the same molecular subtype in BRCA present different clinical outcomes. Thus, the tumor heterogeneity in BRCA might be involved in the poor performance (Figure S1) in our study.

DL-based models are useful to predict the potency of a new drug, to a new cell line. While traditional drug responsive models usually separate models for individual drugs, DL-based models have constructed single models for all drugs from the pharmacogenomics databases. When separate models for either the new drug or the new cell line are unavailable, traditional drug responsive models do not work. However, in such case, DL models can work since a vector concatenating mutation status of the new cell line and chemical properties of the new drug can be fed into the models as input. Subsequently, the DL models can generate the predicted responsiveness in the case.

In this study, there are limitations. GoogLeNet was originally proposed as an image prediction model, and its application to a drug prediction model showed slightly better performance over the ML baseline model. However, in our study, we did not modify the architecture of auxiliary classifiers and inception modules in the original GoogLeNet. In the GoogLeNet application to this field, auxiliary classifiers and inception modules need to be modified for future study. As the second limitation, a simple architecture, autoendcoders were not considered. This is due to the fact that autoencoder-based approaches showed moderate performances. For example, Dr VAE, indicated AUROCs ranging from 0.56 to 0.84 [23]. The third limitation in our study is that the class was assigned by using equal class intervals, resulting in class imbalance. In addition, the subsequent OVR approach may be reflected into the better performance on classes 0 and 2 compared to class 1 (Figure 3A). An alternative method searching for optimal class intervals should be inspected in future.

In conclusion, our comparisons suggest that the CNN models are practical tools in this new era of “precision medicine.”

## 4. Materials and Methods

### 4.1. Training and Test Datasets, and Molecular Fingerprints

Profiling data of cell lines, molecular properties of drugs, and natural logarithms of drug’s half-maximal inhibitory concentration (IC50) data (henceforth, *ln* (IC50)) between cell lines and drugs [53], used for training and testing models (GoogLeNet and DeepIC50), were provided by Genomics in Drug Sensitivity in Cancer (GDSC, https://www.cancerrxgene.org, accessed on 19 July 2021) [34].

The cell line mutation data was given by COSMIC Cell Line Project (CCLP, https://cancer.sanganger.ac.uk, accessed on 19 July 2021) [35]. Cell line mutation profiles (CMPs) are provided by CCLP including 21,213 genomic position data [35].

We obtained molecular properties of drugs, in GDSC, by using the PaDEL molecular fingerprint descriptor software [54] and it included 6543 features. The features consist of PubChem fingerprint (molecular fragments information) [55], molecular weight, and lipophilicity (i.e., XlogP) [54]. After all processes of data preparing, we divided all of this dataset into ratios of 8 to 2, for training and test sets, respectively.

As another independent test set, GC cell line data were obtained from the Cancer Cell Line Encyclopedia (CCLE) [33]. The GC cell line data include 2814 cell line–IC50 pairs for 153 cancer cell lines and 24 drugs.

### 4.2. An Independent Dataset for Clinical Follow-Up in Breast Cancer Patients

For independent validation in real patients, we obtained the TCGA-BRCA breast cancer patient dataset [36] that included genomic mutation data, clinical follow-up, and chemotherapeutics for the individual patients. The TCGA dataset was obtained from the GDC legacy archive (https://portal.gdc.cancer.gov, accessed on 19 July 2021). It included clinical follow-up information and genomic mutation data for 10 chemotherapeutics for 322 patients. In the TCGA-BRCA dataset, the clinical follow-up had response information for treatment measures. The values for the follow-up belonged to ‘complete response’, ‘partial response’, ‘stable disease’, and ‘clinical progress disease’. Thus, we considered the follow-up as readout of responsiveness. The value in the follow-up of each patient was assigned to three classes: ‘complete response’ as class 0 (high responsiveness); both ‘partial response’ and ‘stable disease’ as class 1 (intermediate responsiveness); and ‘clinical progress disease’ as class 2 (low responsiveness) [56]. We also calculated the molecular properties feature of 10 chemotherapeutic data (including doxorubicin, 5-fluorouracil, paclitaxel, docetaxel, demcitabine, tamoxifen, vinorelbine, methotrexate, cisplatin, vinblastine) using PaDEL [54].

### 4.3. GoogLeNet Model Construction

The structure of GoogLeNet consists of several Inception-v1 modules and two auxiliary classifiers. The GoogLeNet model is a deep CNN model with a total of 22 layers (Table S6) [41]. GoogLeNet has several advantages compared to the previous CNN models such as AlexNet. First, it reduced the amount of computation by using the Inception module, and secondly, global average pooling instead of using a fully connected layer was achieved. Through the processes, the efficiency of calculation was increased by reducing the number of parameters, and finally, the vanishing gradient caused by 22 layers was alleviated by applying the auxiliary classifier twice to reflect the learning loss [41,57]. Our GoogLeNet was constructed using Keras 2.3.1, along with the activation function Rectified Linear Unit (ReLu), and the Stochastic Gradient Descent (SGD) optimizer. The main loss and the auxiliary classifier losses 1 and 2 were set to ratios of 1:0.3:0.3, respectively. We trained the GoogLeNet model with 250 epochs and batch size of 300 in the GDSC training set. The value of *ln* (IC50) for the cancer cell line of the drug was divided into three classes: class 0 (high responsiveness), class 1 (intermediate responsiveness), and class 2 (low responsiveness).

### 4.4. Performance Comparisons of GoogLeNet, DeepIC50, and LASSO

The performance of GoogLeNet (inception-v1) [41] was compared based on the predictions by the AlexNet model, DeepIC50 [28] (Table S7. for architecture). The LASSO model [58] was used as a baseline model. 

We used R version 3.5.2 to lower the decimal point and then convert the values to binary for drug functionality. There were 194,750 cell line–drug *ln* (IC50) pairs present in GDSC dataset. We generated a one-dimensional vector for each pair by concatenating the CMPs and drug information. A total of 160,375 input vectors were obtained. By evenly dividing the *ln* (IC50) ranges, the 160,375 *ln* (IC50) values were classified into three classes. Class 0 (high responsiveness) was assigned to *ln* (IC50) less than 2.36; class 1 (intermediate responsiveness) was assigned to *ln* (IC50) between 2.36 and 5.26; and class 2 was assigned to *ln* (IC50) greater than 5.26 (low responsiveness). Subsequently, the 160,375 cases were split into two sets: the training set (128,300 cases) and the test set (32,075 cases). As another independent test set, GC cell line dataset from CCLE was processed in the same way as the GDSC dataset. The total number of GC cell line test sets in CCLE was of 2814 cases.

Due to the multi-class problem in this study, we calculated micro- and macro-average AUROCs by utilizing one-versus-rest (OVR) classifications to compare the performances of DeepIC50 and GoogLeNet [28,59]. In other words, we used micro-average and macro-average AUROCs for comparing the models, because our goal was a three-class, not a binary-class prediction. The two CNN models used the training and validation sets which were split from the GDSC data. Another independent test set was obtained from the CCLE. The scikit-learn python package was used for the calculation.

For macro-average AUROC, three OVR classifications were obtained, due to the three-class problem in this study, resulting in three False Positive Rates (FPRs) and three True Positive Rates (TPRs). Given a TPR in the horizontal axis in the receiver operating characteristic (ROC) curves, the average of the three FPRs corresponding to the TPRs was plotted. Then, the area under of ROC curves (AUROC, or AUC) was obtained as macro-average AUROC [59].

For micro-average AUROC, for case k (k = 1,…,n; n: the number of total cases) in a test set, its real class were one-hot encoded to vector ***y****_k_*: (1,0,0), (0,1,0), and (0,0,1) for classes 0, 1, and 2, respectively. Its predicted score vector ***s****_k_* for case k, by the models, was composed of three elements: the predicted scores of classes 0, 1, and 2 in order. The concatenated vectors **y** and **s** were defined such that ***y*** = ***y***_1_|| ***y***_2_ || … || ***y****_k_* || … || ***y****_n_*, ***s*** = ***s***_1_|| ***s***_2_ || … || ***s****_k_* || … || ***s****_n_*, where operator “||” concatenates its neighbor two vectors. The scikit-learn python package took **y** and **s**, as input, to report micro-average AUROCs [28,47,49,60].

### 4.5. Application of the Local Interpretable Model-Agnostic Explanation (LIME) to the TCGA-BRCA Dataset

To identify important features in GoogLeNet for the TCGA-BRCA dataset, we utilized the local interpretable model-agnostic explanation (LIME) [50], which was provided in the python lime package. All the parameters were set by default with the number of explainable features setting to 100.

## Data Availability

The GoogLeNet codes are available at https://github.com/labnams/GoogLeNet_Drug.

## Supplementary Materials

The following are available online at https://www.mdpi.com/article/10.3390/ijms22147721/s1, Table S1. The three models’ MCC values in the GDSC test set. Table S2: Confusion matrix of GoogLeNet in the GDSC test set. Table S3: Confusion matrix of GoogLeNet in the GC cell line test set. Table S4: Confusion matrix of GoogLeNet in the TCGA-BRCA patient dataset. Table S5. Comparison of AUROC differences of the two CNN models in contrast with LASSO baseline model in the TCGA-BRCA dataset. Table S6: Our implementation of GoogLeNet for drug responsiveness prediction. Table S7. Model architecture of DeepIC50. Figure S1: Application of the three models to the TCGA-BRCA patient dataset.

## References

[1] Ashley E.A. (2016). Towards precision medicine. Nat. Rev. Genet..

[2] Garraway L.A., Verweij J., Ballman K.V. (2013). Precision Oncology: An Overview. J. Clin. Oncol..

[3] Hodson R. (2016). Precision medicine. Nature.

[4] Chambliss A.B., Chan D.W. (2016). Precision medicine: From pharmacogenomics to pharmacoproteomics. Clin. Proteom..

[5] Filipp F.V. (2017). Precision medicine driven by cancer systems biology. Cancer Metastasis Rev..

[6] Ghasemi M., Nabipour I., Omrani A., Alipour Z., Assadi M. (2016). Precision medicine and molecular imaging: New targeted approaches toward cancer therapeutic and diagnosis. Am. J. Nucl. Med. Mol. Imaging.

[7] Shoemaker R.H. (2006). The NCI60 human tumour cell line anticancer drug screen. Nat. Rev. Cancer.

[8] Weinshilboum R.M., Wang L. (2017). Pharmacogenomics: Precision Medicine and Drug Response. Mayo Clin. Proc..

[9] Curtis C., Shah S.P., Chin S.-F., Turashvili G., Rueda O.M., Dunning M.J., Speed D., Lynch A.G., Samarajiwa S., Yuan Y. (2012). The genomic and transcriptomic architecture of 2000 breast tumours reveals novel subgroups. Nature.

[10] Muzny D.M., Bainbridge M.N., Chang K., Dinh H.H., Drummond J.A., Fowler G., Kovar C.L., Lewis L.R., Morgan M.B., Newsham I.F. (2012). Comprehensive molecular characterization of human colon and rectal cancer. Nature.

[11] Shah S.P., Roth A., Goya R., Oloumi A., Ha G., Zhao Y., Turashvili G., Ding J., Tse K., Haffari G. (2012). The clonal and mutational evolution spectrum of primary triple-negative breast cancers. Nature.

[12] Yuan R., Chen S., Wang Y. (2020). Computational Prediction of Drug Responses in Cancer Cell Lines From Cancer Omics and Detection of Drug Effectiveness Related Methylation Sites. Front. Genet..

[13] Falzone L., Salomone S., Libra M. (2018). Evolution of Cancer Pharmacological Treatments at the Turn of the Third Millennium. Front. Pharm..

[14] Jackson A.L., Loeb L.A. (2001). The contribution of endogenous sources of DNA damage to the multiple mutations in cancer. Mutat. Res./Fundam. Mol. Mech. Mutagenesis.

[15] Lawrence M.S., Stojanov P., Polak P., Kryukov G.V., Cibulskis K., Sivachenko A., Carter S.L., Stewart C., Mermel C.H., Roberts S.A. (2013). Mutational heterogeneity in cancer and the search for new cancer-associated genes. Nature.

[16] Loeb L.A., Loeb K.R., Anderson J.P. (2003). Multiple mutations and cancer. Proc. Natl. Acad. Sci. USA.

[17] Stephens P.J., Tarpey P.S., Davies H., Van Loo P., Greenman C., Wedge D.C., Nik-Zainal S., Martin S., Varela I., Bignell G.R. (2012). The landscape of cancer genes and mutational processes in breast cancer. Nature.

[18] Dagogo-Jack I., Shaw A.T. (2018). Tumour heterogeneity and resistance to cancer therapies. Nat. Rev. Clin. Oncol..

[19] Seyhan A.A., Carini C. (2019). Are innovation and new technologies in precision medicine paving a new era in patients centric care?. J. Transl. Med..

[20] Geeleher P., Cox N.J., Huang R.S. (2014). Clinical drug response can be predicted using baseline gene expression levels and in vitrodrug sensitivity in cell lines. Genome Biol..

[21] Li M., Wang Y., Zheng R., Shi X., Li Y., Wu F.X., Wang J. (2021). DeepDSC: A Deep Learning Method to Predict Drug Sensitivity of Cancer Cell Lines. IEEE/ACM Trans. Comput. Biol. Bioinform..

[22] Preuer K., Lewis R.P.I., Hochreiter S., Bender A., Bulusu K.C., Klambauer G. (2017). DeepSynergy: Predicting anti-cancer drug synergy with Deep Learning. Bioinformatics.

[23] Rampášek L., Hidru D., Smirnov P., Haibe-Kains B., Goldenberg A. (2019). Dr.VAE: Improving drug response prediction via modeling of drug perturbation effects. Bioinformatics.

[24] Sharifi-Noghabi H., Zolotareva O., Collins C.C., Ester M. (2019). MOLI: Multi-omics late integration with deep neural networks for drug response prediction. Bioinformatics.

[25] Ding M.Q., Chen L., Cooper G.F., Young J.D., Lu X. (2018). Precision Oncology beyond Targeted Therapy: Combining Omics Data with Machine Learning Matches the Majority of Cancer Cells to Effective Therapeutics. Mol. Cancer Res..

[26] Baptista D., Ferreira P.G., Rocha M. (2020). Deep learning for drug response prediction in cancer. Brief. Bioinform..

[27] Adam G., Rampášek L., Safikhani Z., Smirnov P., Haibe-Kains B., Goldenberg A. (2020). Machine learning approaches to drug response prediction: Challenges and recent progress. NPJ Precis. Oncol..

[28] Joo M., Park A., Kim K., Son W.-J., Lee H.S., Lim G., Lee J., Lee D.H., An J., Kim J.H. (2019). A Deep Learning Model for Cell Growth Inhibition IC50 Prediction and Its Application for Gastric Cancer Patients. Int. J. Mol. Sci..

[29] Liu P., Li H., Li S., Leung K.-S. (2019). Improving prediction of phenotypic drug response on cancer cell lines using deep convolutional network. BMC Bioinform..

[30] Cortés-Ciriano I., Bender A. (2019). KekuleScope: Prediction of cancer cell line sensitivity and compound potency using convolutional neural networks trained on compound images. J. Cheminform..

[31] Chang Y., Park H., Yang H.-J., Lee S., Lee K.-Y., Kim T.S., Jung J., Shin J.-M. (2018). Cancer Drug Response Profile scan (CDRscan): A Deep Learning Model That Predicts Drug Effectiveness from Cancer Genomic Signature. Sci. Rep..

[32] Lamb J., Crawford E.D., Peck D., Modell J.W., Blat I.C., Wrobel M.J., Lerner J., Brunet J.-P., Subramanian A., Ross K.N. (2006). The Connectivity Map: Using Gene-Expression Signatures to Connect Small Molecules, Genes, and Disease. Science.

[33] Barretina J., Caponigro G., Stransky N., Venkatesan K., Margolin A.A., Kim S., Wilson C.J., Lehár J., Kryukov G.V., Sonkin D. (2012). The Cancer Cell Line Encyclopedia enables predictive modelling of anticancer drug sensitivity. Nature.

[34] Yang W., Soares J., Greninger P., Edelman E.J., Lightfoot H., Forbes S., Bindal N., Beare D., Smith J.A., Thompson I.R. (2012). Genomics of Drug Sensitivity in Cancer (GDSC): A resource for therapeutic biomarker discovery in cancer cells. Nucleic Acids Res..

[35] Forbes S.A., Beare D., Boutselakis H., Bamford S., Bindal N., Tate J., Cole C.G., Ward S., Dawson E., Ponting L. (2016). COSMIC: Somatic cancer genetics at high-resolution. Nucleic Acids Res..

[36] Grossman R.L., Heath A.P., Ferretti V., Varmus H.E., Lowy D.R., Kibbe W.A., Staudt L.M. (2016). Toward a Shared Vision for Cancer Genomic Data. N. Engl. J. Med..

[37] Shinde P.P., Shah S. A Review of Machine Learning and Deep Learning Applications. Proceedings of the 2018 Fourth International Conference on Computing Communication Control and Automation (ICCUBEA).

[38] Vaz J.M., Balaji S. (2021). Convolutional neural networks (CNNs): Concepts and applications in pharmacogenomics. Mol. Divers..

[39] Lecun Y., Bottou L., Bengio Y., Haffner P. (1998). Gradient-based learning applied to document recognition. Proc. IEEE.

[40] Véstias M.P. (2019). A Survey of Convolutional Neural Networks on Edge with Reconfigurable Computing. Algorithms.

[41] Szegedy C., Wei L., Yangqing J., Sermanet P., Reed S., Anguelov D., Erhan D., Vanhoucke V., Rabinovich A. Going deeper with convolutions. Proceedings of 2015 IEEE Conference on Computer Vision and Pattern Recognition (CVPR).

[42] Williams A.M., Liu Y., Regner K.R., Jotterand F., Liu P., Liang M. (2018). Artificial intelligence, physiological genomics, and precision medicine. Physiol. Genom..

[43] Sheng J., Li F., Wong S.T.C. (2015). Optimal Drug Prediction From Personal Genomics Profiles. IEEE J. Biomed. Health Inform..

[44] Johnson K.B., Wei W.-Q., Weeraratne D., Frisse M.E., Misulis K., Rhee K., Zhao J., Snowdon J.L. (2021). Precision Medicine, AI, and the Future of Personalized Health Care. Clin. Transl. Sci..

[45] Chiu Y.-C., Chen H.-I.H., Zhang T., Zhang S., Gorthi A., Wang L.-J., Huang Y., Chen Y. (2019). Predicting drug response of tumors from integrated genomic profiles by deep neural networks. BMC Med. Genom..

[46] Sokolova M., Lapalme G. (2009). A systematic analysis of performance measures for classification tasks. Inf. Process. Manag..

[47] Kautz T., Eskofier B.M., Pasluosta C.F. (2017). Generic performance measure for multiclass-classifiers. Pattern Recognit..

[48] Hand D.J., Till R.J. (2001). A Simple Generalisation of the Area Under the ROC Curve for Multiple Class Classification Problems. Mach. Learn..

[49] Jin H., Ling C.X. (2005). Using AUC and accuracy in evaluating learning algorithms. IEEE Trans. Knowl. Data Eng..

[50] Ribeiro M.T., Singh S., Guestrin C. “Why Should I Trust You?”: Explaining the Predictions of Any Classifier. Proceedings of the 22nd ACM SIGKDD International Conference on Knowledge Discovery and Data Mining.

[51] Lüönd F., Tiede S., Christofori G. (2021). Breast cancer as an example of tumour heterogeneity and tumour cell plasticity during malignant progression. Br. J. Cancer.

[52] Turashvili G., Brogi E. (2017). Tumor Heterogeneity in Breast Cancer. Front. Med..

[53] Gary W.C., Zhengyin Y., Wensheng L., John A.M. (2012). The IC50 Concept Revisited. Curr. Top. Med. Chem..

[54] Yap C.W. (2011). PaDEL-descriptor: An open source software to calculate molecular descriptors and fingerprints. J. Comput. Chem..

[55] Fernández-de Gortari E., García-Jacas C.R., Martinez-Mayorga K., Medina-Franco J.L. (2017). Database fingerprint (DFP): An approach to represent molecular databases. J. Cheminform..

[56] Subbiah V., Chuang H.H., Gambhire D., Kairemo K. (2017). Defining Clinical Response Criteria and Early Response Criteria for Precision Oncology: Current State-of-the-Art and Future Perspectives. Diagnostics (Basel).

[57] Qian Z., Hayes T.L., Kafle K., Kanan C. (2020). Do We Need Fully Connected Output Layers in Convolutional Networks?. arXiv.

[58] You J., McLeod R.D., Hu P. (2019). Predicting drug-target interaction network using deep learning model. Comput. Biol. Chem..

[59] Krawczyk B., Galar M., Woźniak M., Bustince H., Herrera F. (2018). Dynamic ensemble selection for multi-class classification with one-class classifiers. Pattern Recognit..

[60] Fawcett T. (2006). An introduction to ROC analysis. Pattern Recognit. Lett..

