# Supplementary material for: Performance Comparisons of AlexNet and GoogLeNet in Cell Growth Inhibition IC50 Prediction
Source: Int J Mol Sci. 2021 Jul 19;22(14):7721. doi: 10.3390/ijms22147721 (PMC8305019; doi:10.3390/ijms22147721)

Supporting Information

Performance comparisons of AlexNet and GoogLeNet in cell growth inhibition IC50 prediction

Lee *et al*.

Contents

Table S1 through S7
Figure S1

Table S1. The three models' MCC values in the GDSC test set.

OVR classification	GoogLeNet	DeepIC50	LASSO
**Class 0 vs. others**	0.504256027	0.545457535	0.557941156
**Class 1 vs. others**	0.411364275	0.454712104	0.392195306
**Class 2 vs. others**	0.348978850	0.386608536	0.281037632

Table S2. Confusion matrix of GoogLeNet in the GDSC test set.

Observed \ Predicted	class 0	class 1	class 2
**class 0**	2060	894	0
**class 1**	736	25204	668
**class 2**	0	1145	1368

Table S3. Confusion matrix of GoogLeNet in the GC cell line test set.

Observed \ Predicted	class 0	class 1	class 2
**class 0**	171	372	1
**class 1**	142	1954	174
**class 2**	0	0	0

Table S4. Confusion matrix of GoogLeNet in the TCGA-BRCA patient dataset.

Observed \ Predicted	class 0	class 1	class 2
**class 0**	137	143	1
**class 1**	14	8	0
**class 2**	6	13	0

Table S5. Comparison of AUROC differences of the two CNN models in contrast with LASSO baseline model in the TCGA-BRCA dataset. '△' indicates the difference in contrast with LASSO.

	**△ micro-average AUROC**	**△ macro-average AUROC**
LASSO	0.00	0.00
GoogLeNet	0.03	0.09
DeepIC50	-0.03	0.02

Table S6. Our implementation of GoogLeNet for drug responsiveness prediction. The GoogLeNet structure uses specific modules called 'Inception' and 'Auxiliary classifier'. The 'module type' column represents that type of specific modules, the 'Param #' column represents number of parameters, respectively.

Layer (type)	Output Shape	Param #	module type
input_1 (InputLayer)	(167x167x1)	0	
conv2d_1 (Conv2D)	(84x84x64)	3200	
max_pooling2d_1 (MaxPooling2D)	(42x42x64)	0	
local_response_normalization_1	(42x42x64)	0	
conv2d_2 (Conv2D)	(42x42x64)	4160	
conv2d_3 (Conv2D)	(42x42x192)	110784	
local_response_normalization_2	(42x42x192)	0	
max_pooling2d_2 (MaxPooling2D)	(21x21x192)	0	
conv2d_5 (Conv2D)	(21x21x96)	18528	Inception (3a)
conv2d_7 (Conv2D)	(21x21x16)	3088	Inception (3a)
max_pooling2d_3 (MaxPooling2D)	(21x21x192)	0	Inception (3a)
conv2d_4 (Conv2D)	(21x21x64)	12352	Inception (3a)
conv2d_6 (Conv2D)	(21x21x128)	110720	Inception (3a)
conv2d_8 (Conv2D)	(21x21x32)	12832	Inception (3a)
conv2d_9 (Conv2D)	(21x21x32)	6176	Inception (3a)
concatenate_1 (Concatenate)	(21x21x256)	0	Inception (3a)
conv2d_11 (Conv2D)	(21x21x128)	32896	Inception (3b)
conv2d_13 (Conv2D)	(21x21x32)	8224	Inception (3b)
max_pooling2d_4 (MaxPooling2D)	(21x21x256)	0	Inception (3b)
conv2d_10 (Conv2D)	(21x21x128)	32896	Inception (3b)
conv2d_12 (Conv2D)	(21x21x192)	221376	Inception (3b)
conv2d_14 (Conv2D)	(21x21x96)	76896	Inception (3b)
conv2d_15 (Conv2D)	(21x21x64)	16448	Inception (3b)
concatenate_2 (Concatenate)	(21x21x480)	0	Inception (3b)
max_pooling2d_5 (MaxPooling2D)	(11x11x480)	0	
conv2d_17 (Conv2D)	(11x11x96)	46176	Inception (4a)
conv2d_19 (Conv2D)	(11x11x16)	7696	Inception (4a)
max_pooling2d_6 (MaxPooling2D)	(11x11x480)	0	Inception (4a)
conv2d_16 (Conv2D)	(11x11x192)	92352	Inception (4a)
conv2d_18 (Conv2D)	(11x11x208)	179920	Inception (4a)
conv2d_20 (Conv2D)	(11x11x48)	19248	Inception (4a)
conv2d_21 (Conv2D)	(11x11x64)	30784	Inception (4a)
concatenate_3 (Concatenate)	(11x11x512)	0	Inception (4a)
conv2d_23 (Conv2D)	(11x11x112)	57456	Inception (4b)
conv2d_25 (Conv2D)	(11x11x24)	12312	Inception (4b)

max_pooling2d_7 (MaxPooling2D)	(11x11x512)	0	Inception (4b)
conv2d_22 (Conv2D)	(11x11x160)	82080	Inception (4b)
conv2d_24 (Conv2D)	(11x11x224)	226016	Inception (4b)
conv2d_26 (Conv2D)	(11x11x64)	38464	Inception (4b)
conv2d_27 (Conv2D)	(11x11x64)	32832	Inception (4b)
concatenate_4 (Concatenate)	(11x11x512)	0	Inception (4b)
conv2d_29 (Conv2D)	(11x11x128)	65664	Inception (4c)
conv2d_31 (Conv2D)	(11x11x24)	12312	Inception (4c)
max_pooling2d_8 (MaxPooling2D)	(11x11x512)	0	Inception (4c)
conv2d_28 (Conv2D)	(11x11x128)	65664	Inception (4c)
conv2d_30 (Conv2D)	(11x11x256)	295168	Inception (4c)
conv2d_32 (Conv2D)	(11x11x64)	38464	Inception (4c)
conv2d_33 (Conv2D)	(11x11x64)	32832	Inception (4c)
concatenate_5 (Concatenate)	(11x11x512)	0	Inception (4c)
conv2d_35 (Conv2D)	(11x11x144)	73872	Inception (4d)
conv2d_37 (Conv2D)	(11x11x32)	16416	Inception (4d)
max_pooling2d_9 (MaxPooling2D)	(11x11x512)	0	Inception (4d)
conv2d_34 (Conv2D)	(11x11x112)	57456	Inception (4d)
conv2d_36 (Conv2D)	(11x11x288)	373536	Inception (4d)
conv2d_38 (Conv2D)	(11x11x64)	51264	Inception (4d)
conv2d_39 (Conv2D)	(11x11x64)	32832	Inception (4d)
concatenate_6 (Concatenate)	(11x11x528)	0	Inception (4d)
conv2d_41 (Conv2D)	(11x11x160)	84640	Inception (4e)
conv2d_43 (Conv2D)	(11x11x32)	16928	Inception (4e)
max_pooling2d_10 (MaxPooling2D)	(11x11x528)	0	Inception (4e)
conv2d_40 (Conv2D)	(11x11x256)	135424	Inception (4e)
conv2d_42 (Conv2D)	(11x11x320)	461120	Inception (4e)
conv2d_44 (Conv2D)	(11x11x128)	102528	Inception (4e)
conv2d_45 (Conv2D)	(11x11x128)	67712	Inception (4e)
concatenate_7 (Concatenate)	(11x11x832)	0	Inception (4e)
max_pooling2d_11 (MaxPooling2D)	(6x6x832)	0	
conv2d_47 (Conv2D)	(6x6x160)	133280	Inception (5a)
conv2d_49 (Conv2D)	(6x6x32)	26656	Inception (5a)
max_pooling2d_12 (MaxPooling2D)	(6x6x832)	0	Inception (5a)
conv2d_46 (Conv2D)	(6x6x256)	213248	Inception (5a)
conv2d_48 (Conv2D)	(6x6x320)	461120	Inception (5a)
conv2d_50 (Conv2D)	(6x6x128)	102528	Inception (5a)
conv2d_51 (Conv2D)	(6x6x128)	106624	Inception (5a)
concatenate_8 (Concatenate)	(6x6x832)	0	Inception (5a)
conv2d_53 (Conv2D)	(6x6x192)	159936	Inception (5b)

conv2d_55 (Conv2D)	(6x6x48)	39984	Inception (5b)
max_pooling2d_13 (MaxPooling2D)	(6x6x832)	0	Inception (5b)
conv2d_52 (Conv2D)	(6x6x384)	319872	Inception (5b)
conv2d_54 (Conv2D)	(6x6x384)	663936	Inception (5b)
conv2d_56 (Conv2D)	(6x6x128)	153728	Inception (5b)
conv2d_57 (Conv2D)	(6x6x128)	106624	Inception (5b)
average_pooling2d_1 (AveragePooling2D)	(3x3x512)	0	Inception (5b)
average_pooling2d_2 (AveragePooling2D)	(3x3x528)	0	Inception (5b)
concatenate_9 (Concatenate)	(6x6x1024)	0	Inception (5b)
conv2d_58 (Conv2D)	(3x3x128)	65664	average pooling
conv2d_59 (Conv2D)	(3x3x128)	67712	average pooling
global_average_pooling2d_1 (GlobalAveragePooling2D)	(1024)	0	average pooling
flatten_1 (Flatten)	(1152)	0	auxiliary classifier (4a)
flatten_2 (Flatten)	(1152)	0	auxiliary classifier (4d)
dropout_1 (Dropout)	(1024)	0	main classifier
dense_2 (Dense)	(1024)	1180672	auxiliary classifier (4a)
dense_3 (Dense)	(1024)	1180672	auxiliary classifier (4d)
dense_1 (Dense)	(1000)	1025000	main classifier
dropout_2 (Dropout)	(1024)	0	auxiliary classifier (4a)
dropout_3 (Dropout)	(1024)	0	auxiliary classifier (4d)
main_classifier (Dense)	(3)	3003	main classifier
auxiliary_4a (Dense)	(3)	3075	auxiliary classifier (4a)
auxiliary_4d (Dense)	(3)	3075	auxiliary classifier (4d)

Total Parameters : 9,496,153

Table S7. Model architecture of DeepIC50. The 'Param #' column represents number of parameters, respectively.

**Layer (type)**	**Output Shape**	**Param #**
input_1 (InputLayer)	(27889×1)	0
conv1d_1 (Conv1D)	(27889×16)	192
batch_normalization_1 (BatchNormalization)	(27889×16)	64
conv1d_2 (Conv1D)	(27889×16)	2832
batch_normalization_2 (BatchNormalization)	(27889×16)	64
max_pooling1d_1 (MaxPooling1D)	(13945×16)	0
conv1d_3 (Conv1D)	(13945×32)	5664
batch_normalization_3 (BatchNormalization)	(13945×32)	128
conv1d_4 (Conv1D)	(13945×32)	11296
max_pooling1d_2 (MaxPooling1D)	(6973×32)	0
batch_normalization_4 (BatchNormalization)	(6973×32)	128
conv1d_5 (Conv1D)	(6973×64)	22592
batch_normalization_5 (BatchNormalization)	(6973×64)	256
max_pooling1d_3 (MaxPooling1D)	(3487×64)	0
conv1d_6 (Conv1D)	(3487×64)	45120
batch_normalization_6 (BatchNormalization)	(3487×64)	256
max_pooling1d_4 (MaxPooling1D)	(1744×64)	0
flatten_1 (Flatten)	(111616)	0
dense_1 (Dense)	(1024)	114295808
batch_normalization_7 (BatchNormalization)	(1024)	4096
dropout_1 (Dropout)	(1024)	0
dense_2 (Dense)	(2048)	2099200
batch_normalization_8 (BatchNormalization)	(2048)	8192
dropout_2 (Dropout)	(2048)	8392704
dense_3 (Dense)	(4096)	16384
batch_normalization_9 (BatchNormalization)	(4096)	0
dropout_3 (Dropout)	(4096)	8390656
dense_4 (Dense)	(2048)	8192
batch_normalization_10 (BatchNormalization)	(2048)	0
dropout_4 (Dropout)	(2048)	2098176
dense_5 (Dense)	(1024)	4096
batch_normalization_11 (BatchNormalization)	(1024)	0
dropout_5 (Dropout)	(1024)	3075
dense_6 (Dense)	(3)	135409171

Total Parameters : 135,409,171

Figure S1. Application of the three models to the TCGA-BRCA Patient Dataset.
(A) ROC curves representing the performance of GoogLeNet for the TCGA-BRCA patient dataset. **(B)** Micro-average ROC curves comparing the prediction performances of two CNN models and LASSO for the TCGA-BRCA patient dataset. **(C)** Macro-average ROC curves comparing the prediction performances of two CNN models and LASSO for the TCGA-BRCA patient dataset.

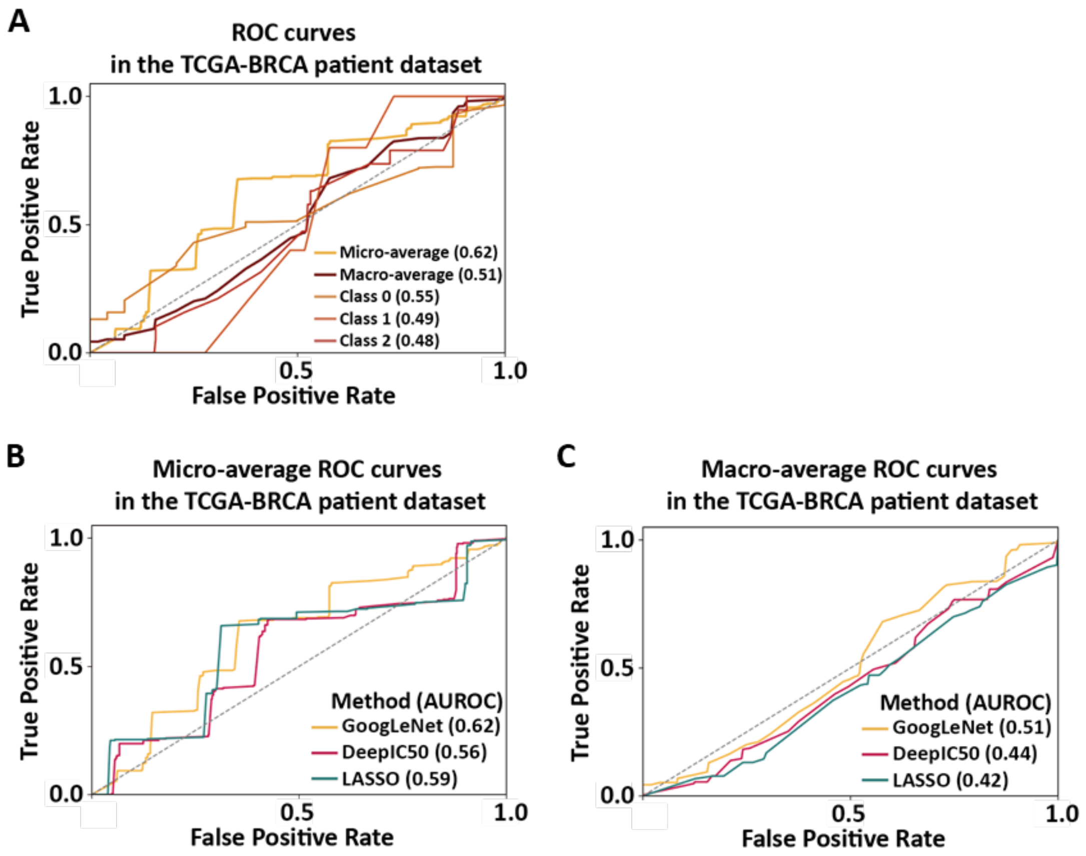

Supplement: Supplementary file 1 [file ijms-22-07721-s001.zip › ijms-1238276-supplementary.pdf]
